# Nipah: An Interesting stance

**DOI:** 10.15171/hpp.2020.03

**Published:** 2020-01-28

**Authors:** Ruchi D. Raval, Mansi Mehta

**Affiliations:** ^1^Department of Periodontics & Implantology, Manubhai Patel Dental College, Munjmahuda, Vadodara 390011, India; ^2^Dr. Bristol Dental, Bristol, Connecticut, USA

**Keywords:** Communicable disease, Epidemics, Henipavirus infections, Nipah Virus, Review

## Abstract

Nipah instead was one of the most fatal outbreaks of diseases in the mankind which was initially assumed as Japanese encephalitis. A multidisciplinary exploration was done at several levels of microbiology, histopathology and genetics which led to the discovery of a new paramyxovirus named Nipah virus (NiV). The disease was primarily identified in Malaysia in 1998 and named after a village, Sungai Nipah. The main mode of transmission in the Malaysian outbreaks was thought to be the consumption of bat’s dropping, urine and fruit partially eaten by pigs. In Bangladesh and northeast India, the virus was directly transmitted from bats to human through consumption of raw date palm juice. To limit the epidemic, coordinated efforts by health care providers have become mandatory. This article gives a note about the NiV, its infection and on-going researches on its management strategies. Data were collected using electronic media consisting of articles, books and websites.

## Introduction

Nipah instead was one of the most fatal outbreaks of diseases in the mankind which was initially erroneously assumed as Japanese encephalitis. A multidisciplinary exploration was done at several levels of microbiology, histopathology and genetics which led to the discovery of a new paramyxovirus named Nipah virus (NiV). The disease was primarily identified in Malaysia in 1998 and named after a village, Sungai Nipah.^1^ It subsequently extended to the wider belts of Asia and Africa with mortality as high as 70%. The main mode of transmission in the Malaysian outbreaks was thought to be the consumption of bat’s dropping, urine and fruit partially eaten by pigs in the intensive pig farms in peninsular Malaysia, where fruit trees were planted in pig farms to yield extra income for the farmers.^2^ In Bangladesh and northeast India virus was directly transmitted from bats to human through consumption of raw date palm juice.^3^ To curb the epidemic, a meticulous and coordinated effort by health care providers and veterinary doctors has become mandatory. This article gives a note about the NiV, its infection and on-going researches on its management strategies. Data was collected using electronic media consisting of articles, books and websites.

## Virus

NiV belongs to the subfamily Paramyxovirinae covering the five genera Respiro-, Morbilli-, Rubula-, Avula-, and Henipavirus, and a group of uncategorized viruses so far. The genome is made up of six genes (N, P, M, F, G and L) of nucleoprotein, phosphoprotein, matrix, fusion, glycoprotein and large RNA polymerase and three non-structural proteins which riles on the host innate immune response in vitro.^4^

## Natural host

Pteropid fruit bats, i.e. *Pteropus vampyrus*  (large flying fox), and *Pteropus hypomelanus*  (small flying fox) are the prime pool for the virus which routinely spreads to pigs making it the second most common habitat. Bat being the airborne and outgoing creature can easily transmit the virus with its distribution as wide as in 10 genera and 23 species of bats and other genus.^5^

## Emergence

Nipah is an emerging infectious disease with a natural spread from animals to humans due to their enhanced interface. This exchange of virus could be attributed to forest clearing done to promote agricultural growth, international voyage, trade in wildlife, and other anthropogenic factors.^6-8^ Fire for deforestation produces sulphate and organic carbon particles in haze, leading to reduction in 73%-92% of total light, altering the rain forest and rest of ecosystem.^9^Tang et al. in 1996 accounted that diminished activity of photosynthesis by forest trees could be attributed to 1994 haze event in Malaysia.^10^ The smog considerably condensed flowering and fruiting among orchard fruit trees in southern peninsular Malaysia.

The menace to tropical rainforests of Amazon, Africa and Southeast Asia was caused by agricultural ranching in these areas.^7^Industrialization and dearth of natural resources have lead bats to migrate and make a permanent abode in other farms.^11^ Even the pigs are often transported to the southern parts of Malaysia and Singapore for trade, creating a front for infection in these regions.This loss of foraging habitation for fruitbats, coupled with increasing deforestation, propagated their migration into cultivated orchards and human terrain.^12^

Chief dissimilarities between the outbreaks in Bangladesh and those in Malaysia and Singapore were proposed in epidemiological survey: (1) In all the five independent outbreaks, the NiV has tipped on humans; (2) The outbreaks are seasonal; (3) Virus tipping occurred without domestic animals; (4) Data suggests spread between humans. Genetic mapping of virus between human samples from Bangladesh and Malaysia were distinct.^13^

## Transmission^14^

From bat to human: Generally, the dissemination occurs by consumption of fresh date palm sap, spoiled with bat secretions. Other routes include NiV infected domestic animals which drop the virus to humans during contact.

Human-to-human transmission: Human-to-human transmission was only documented in the Bangladeshi and Indian outbreaks. There was no definite human-to-human transmission in the Malaysian outbreaks; though studies showed serological scars in health care workers.

## The innate immune response to NiV

Very less data is available about the effect of NiV on the innate immune system. However, many in vitro studies have established, that the infected endothelial cells secrete IFN-β, chemokines and cytokines.^15^

## The adaptive immune response to NiV

Research in this subject is still requisite, yet some evidences do show recruitment of immune cell IgG and IgM in the infected patients. Whether the disease is due to incompetent immunity or hyper responsive system is still questionable.^13^

## Clinical features

Signs and symptoms include fever, unsettled stomach, light-headedness and headache. Encephalitis of aggressive nature is frequent and has been associated with elevated mortality index.^16^

## Diagnosis

Investigations for NiV include serum neutralization, enzyme-linked immunosorbent assay (ELISA), polymerase chain reaction (PCR) assays, immunofluorescence assay and virus isolation by cell culture.^17^

## Prevention^14^

Care of domestic animals: Maintaining the sanitary conditions by regularly bathing them, removing excreta, saliva and secretions, cleaning of the pig farms with sodium hypochlorite solution and limiting the migration of the animals from an infected neighbourhood can go a long way in minimizing the zoonotic culprit. Animal health surveillance system should be proactive in identifying the development of new cases and timely notifying preventive and community department.

Public health awareness programs should consider following points:

1. Alleviate the risk of bat-to-human transmission: Keep the fruit farms of date palm sap safe from bats. Maintain a check on the quality with a habit of washing and boiling before consuming juice and fruits.
2. Alleviate the risk of human-to-human transmission: Prevent any physical contact with NiV infected individuals, if at all use personal protective barriers as a shield.
3. Alleviate the risk of animal-to-human transmission: Personal protective barriers should be used while managing sick animals.

## Antiviral treatment

In vitro and animal studies have partly proved the effectiveness of ribavirin in delaying the progression of the disease pathogenesis.^18^ Nevertheless; some historically controlled trials do support the use of ribavirin as the main stay of treatment.^19^

## Vaccination

Several animal studies have confirmed that both the HeV-sG vaccine and the m102.4 human antibody can be used against Niv. The HeV-sG subunit immunogen has been adopted as an effectual vaccine in horses.^20^Nonetheless, its use in developing countries of Southeast Asia is questionable considering its expenditure.

## Conclusion

Nipah is a deadly virus which has taken a toll over the mankind. The infection control team should have thorough perception and conception of the disease. Additional researches need to be diverted towards preventive and management strategies consisting chiefly of vaccines to curb the epidemic.

## Ethical approval

Not Applicable.

## Competing interests

The authors declare that they have no competing interests.

## Funding

None.

## Authors’ contributions

RDR: Concept and design, definition of intellectual content, literature research, clinical studies, experimental studies, data acquisition, data analysis, statistical analysis, manuscript preparation, manuscript editing and manuscript review. MM: Literature research, data acquisition, manuscript editing and manuscript review.

## Acknowledgments

I would like to thank my colleagues for providing support for searching the literature of the article.

## References

[1] Paton NI, Leo YS, Zaki SR, Auchus AP, Lee KE, Ling AE (1999). Outbreak of Nipah-virus infection among abattoir workers in Singapore. Lancet.

[2] Middleton DJ, Westbury HA, Morrissy CJ, van der Heide BM, Russell GM, Braun MA (2002). Experimental Nipah virus infection in pigs and cats. J Comp Pathol.

[3] Khan M, Nahar N, Sultanan R, Hossain M, Gurley ES, Luby S. Understanding bats access to date palm sap: identifying preventative techniques for Nipah virus transmission. New Orleans: American Society of Tropical Medicine and Hygiene, 2008. p. 331.

[4] King AMQ, Adams MJ, Carstens EB, Lefkowitz EJ. Virus Taxonomy: Classification and Nomenclature of Viruses -- Ninth Report of the International Committee on Taxonomy of Viruses. Amsterdam: Academic Press; 2011. P. 1261-91.

[5] Chong HT, Abdullah S, Tan CT (2009). Nipah virus and bats. Neurol Asia.

[6] Field H, Young P, Yob JM, Mills J, Hall L, Mackenzie J (2001). The natural history of Hendra and Nipah viruses. Microbes Infect.

[7] Malingreau JP, Stephens G, Fellows L (1985). Remote sensing of forest fires: Kalimantan and North Borneo in 1982-83. Ambio.

[8] Schwedhelm J. The fire this time. An overview of Indonesia’s forest fire 1997-1998. World Wide Fund for Nature Discussion paper. WWF Indonesia programme; 1998.

[9] Barrie LA, Hoff RM, Daggupaty SM (1981). The influence of mid-latitudinal pollution sources on haze in the Canadian arctic. Atmos Environ.

[10] Yanhong T, Naoki K, Akio F, Awang M (1996). Light reduction by regional haze and its effect on simulated leaf photosynthesis in a tropical forest of Malaysia. For Ecol Manage.

[11] Aziz J, Olson J, Lee OB, Daniels P, Adzhar AB, Bunning M, et al. NiV of animals in Malaysia. In: Abstracts of the XI International Congress of Virology; August 9-13, 1999; Sydney, Australia.

[12] Epstein JH, Field HE, Luby S, Pulliam JR, Daszak P (2006). Nipah virus: impact, origins, and causes of emergence. Curr Infect Dis Rep.

[13] Chong HT, Kamarulzaman A, Tan CT, Goh KJ, Thayaparan T, Kunjapan SR (2001). Treatment of acute Nipah encephalitis with ribavirin. Ann Neurol.

[14] World Health Organization (WHO). The Weekly Epidemiological Record (WER). Available from: http://www.who.int/wer/en/. Accessed October 24, 2018.

[15] Lo MK, Miller D, Aljofan M, Mungall BA, Rollin PE, Bellini WJ (2010). Characterization of the antiviral and inflammatory responses against Nipah virus in endothelial cells and neurons. Virology.

[16] Goh KJ, Tan CT, Chew NK, Tan PS, Kamarulzaman A, Sarji SA (2000). Clinical features of Nipah virus encephalitis among pig farmers in Malaysia. N Engl J Med.

[17] Daniels P, Ksiazek T, Eaton BT (2001). Laboratory diagnosis of Nipah and Hendra virus infections. Microbes Infect.

[18] Freiberg AN, Worthy MN, Lee B, Holbrook MR (2010). Combined chloroquine and ribavirin treatment does not prevent death in a hamster model of Nipah and Hendra virus infection. J Gen Virol.

[19] Chong HT, Kamarulzaman A, Tan CT, Goh KJ, Thayaparan T, Kunjapan SR (2001). Treatment of acute Nipah encephalitis with ribavirin. Ann Neurol.

[20] Bossart KN, Rockx B, Feldmann F, Brining D, Scott D, LaCasse R (2012). A Hendra virus G glycoprotein subunit vaccine protects African green monkeys from Nipah virus challenge. Sci Transl Med.

